# Stress Hyperglycemia in Children and Adolescents as a Prognostic Indicator for the Development of Type 1 Diabetes Mellitus

**DOI:** 10.3389/fped.2021.670976

**Published:** 2021-04-26

**Authors:** Theodoros Argyropoulos, Emmanouil Korakas, Aristofanis Gikas, Aikaterini Kountouri, Stavroula Kostaridou-Nikolopoulou, Athanasios Raptis, Vaia Lambadiari

**Affiliations:** ^1^Department of Paediatrics, Penteli Children's Hospital, Athens, Greece; ^2^Second Department of Internal Medicine and Research Institute, Medical School, Attikon University Hospital, National and Kapodistrian University of Athens, Athens, Greece; ^3^Health Center of Kalivia, Athens, Greece

**Keywords:** stress hyperglycemia, type 1 diabetes mellitus, autoantibodies, inflammation, environmental factors

## Abstract

Hyperglycemia is a common manifestation in the course of severe disease and is the result of acute metabolic and hormonal changes associated with various factors such as trauma, stress, surgery, or infection. Numerous studies demonstrate the association of adverse clinical events with stress hyperglycemia. This article briefly describes the pathophysiological mechanisms which lead to hyperglycemia under stressful circumstances particularly in the pediatric and adolescent population. The importance of prevention of hyperglycemia, especially for children, is emphasized and the existing models for the prediction of diabetes are presented. The available studies on the association between stress hyperglycemia and progress to type 1 diabetes mellitus are presented, implying a possible role for stress hyperglycemia as part of a broader prognostic model for the prediction and prevention of overt disease in susceptible patients.

## Introduction

The term stress hyperglycemia (SH) refers to a transient increase in plasma glucose levels (usually above 150 mg/dl) during acute illness or physical or psychological stress, which subsides when the stressful condition resolves (1). Specifically, according to the latest American Diabetes Association and American Association of Clinical Endocrinologists consensus, stress hyperglycemia is defined as any transient inpatient plasma glucose levels > 140 mg/dl (fasting plasma glucose of >126 mg/dl or random plasma glucose > 200 mg/dl) without evidence of previous diabetes (2). Common causes in children include febrile infections and seizures, trauma, cardiac surgery and burns, and its incidence in the pediatric population admitted to the hospital is quite remarkable. Almost 3.8-5% of non-diabetic children presenting to the emergency department have glucose levels >150 mg/dl, while about 20-35% of critically ill children surpass the cut-off point of 200 mg/dl in the intensive care unit (ICU) (3). In most patients, hyperglycemia is mild to moderate, with blood glucose concentrations ranging between 150 and 299 mg/dl, however, values of 300 mg/dl or higher have also been reported in smaller cohorts, especially in the ICU setting.

Stress hyperglycemia is considered a normal metabolic response to acute stress (4). It develops through complex pathophysiologic pathways, where counter-regulatory hormones such as cortisol, catecholamines, glucagon, and proinflammatory cytokines stimulate glycogenolysis and gluconeogenesis, leading to increased hepatic glucose output and peripheral insulin resistance. Although this condition was initially considered a protective homeostatic response, studies both in children and adults have associated stress hyperglycemia with adverse clinical outcomes and complications (1). In children, a cardinal question has been whether transient hyperglycemia may represent the earliest clinical sign of impaired β-cell function. In the last decades, much has been learned about the pathogenesis and natural history of type 1 diabetes mellitus (T1DM) (5). Substantial research has been conducted to elucidate not only the genetic markers which indicate children at greatest risk, but also environmental factors, perinatal conditions, family history, and other variables which could accelerate or trigger the occurrence of T1DM and, therefore, can be used for prediction and prevention of the disease (6). In this review, the main pathophysiological pathways through which stress hyperglycemia develops will be described, along with the main causes in pediatric patients. Then, we discuss in brief the factors which are currently used for prediction of T1DM, and we present the main studies on the association of stress hyperglycemia with T1DM and its possible role as a predictive factor in children and adolescents.

## Literature Search Strategy

The studies selected for this review were identified using the PubMed, Scopus, and Web of Science electronic databases. We searched for scientific literature published in English up to January 2021. Combinations of the following search terms were applied: “stress hyperglycemia,” “acute hyperglycemia,” “stress hyperglycemia and type 1 diabetes mellitus,” “pediatric hyperglycemia,” “type 1 diabetes screening,” “type 1 diabetes prediction.” Additional references were retrieved from reviewing the references cited in the original articles.

## Stress Hyperglycemia: Mechanisms and Causes

### Pathophysiology of Stress Hyperglycemia

**Figure 1:**
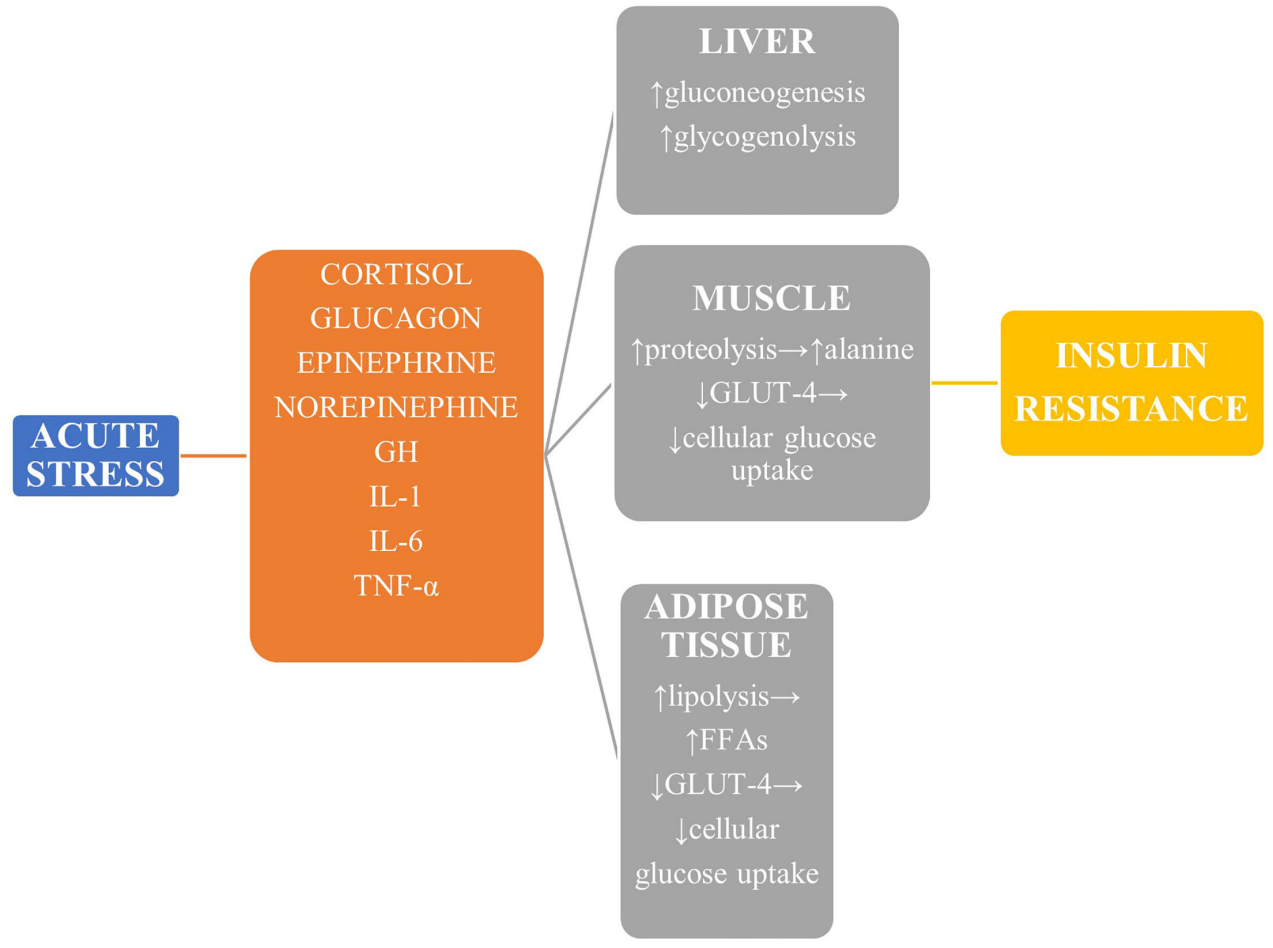
The main mechanisms leading to stress hyperglycemia. Reduced GLUT-4-mediated glucose transport in liver, muscle, and adipose tissue leads to derangement of the insulin-signaling pathway and reduced glucose uptake, while glucose production is upregulated mainly *via* enhanced gluconeogenesis. GH, growth hormone; TNF-α, tumor necrosis factor α; GLUT-4, glucose transporter protein 4.

During stressful conditions, glucose homeostasis is disrupted both in terms of glucose metabolism and insulin sensitivity and secretion (Figure 1). The stress response is mediated by the sympathoadrenal system and the hypothalamic—pituitary—adrenal (HPA) axis (4). The vast increase in counter-regulatory hormones such as catecholamines, growth hormone, cortisol, epinephrine, norepinephrine, and glucagon lead to increased glycogenolysis and gluconeogenesis in a non-insulin-dependent manner. The enhanced muscle degradation due to increased production of growth hormone (GH) and decreased production of insulin-like growth factor-1 (IGF-1) provides liver with alanine to sustain continued gluconeogenesis (7), which is also supported by the kidneys under the effect of catecholamines to a magnitude of even 40% of total glucose production (8). Glucagon, cortisol, and epinephrine are mainly responsible for hepatic insulin resistance, while peripheral insulin resistance is attributed to the derangement of the insulin-signaling pathway in muscles and adipose tissue (9). In addition, reduced skeletal muscle glycogen synthesis leads to impaired non-oxidative glucose disposal (1). Stress hormones decrease the translocation of glucose transporter protein 4 (GLUT-4) to the cell membrane, thus diminishing glucose cellular uptake, and a similar result is exerted by proinflammatory cytokines, mainly interleukin 1 (IL-1), interleukin 6 (IL-6), and tumor necrosis factor α (TNF-α) (10, 11). This diminished insulin-mediated glucose uptake, however, is accompanied by an increase in the whole-body glucose uptake due to the cytokine-mediated upregulation of GLUT-1. Finally, GH and catecholamines induce lipolysis, with the high amounts of circulating free fatty acids contributing to insulin resistance by disrupting insulin signaling and glycogen synthase.

## Stress Hyperglycemia in Pediatric Patients

Stress hyperglycemia has been associated with various diseases in the emergency department. Febrile seizures are among the most commonly reported conditions. In a large study on 1,199 children by Valerio et al., stress hyperglycemia was reported with a three times higher frequency in children with febrile seizures than in other febrile conditions and a five times higher frequency than in seizures without fever (12.9 vs. 4% and 2.4%, respectively) (12). Similarly, in a study by Costea et al. (13), the prevalence of SH among children with febrile seizures was 16.9%, and a positive correlation between SH and duration of the seizure (>15 min), recurrence of the seizure and fever ≥39.5°C was demonstrated, and similar results were demonstrated by Lee et al. (14). On the other hand, in a retrospective study by Levmore-Tamir et al., where 1,245 children with SH were enrolled, the major conditions associated with SH were respiratory (37.8%), neurologic (14.8), and gastrointestinal diseases (14.1%), with trauma and cardiac diseases following (15). Inhaled beta-adrenergic stimulants, which are commonly prescribed in respiratory tract infections, have long been known to cause SH by enhancing gluconeogenesis and glycogenolysis through stimulation of the β2-receptor, especially in the first 2 h after administration (16), and a similar correlation has been found during the administration of adrenaline in children with wheezing (15). Finally, acute diarrhea due to infectious agents such as *E. Coli* and *Vibrio Cholerae* has long been related to stress hyperglycemia, with its prevalence ranging from 9.4 to even 20% in some reports, as a result of a stress response to hypovolemia (17).

Unlike the emergency department, SH in the ICU occurs mainly due to iatrogenic interventions. Mechanical ventilation is a well-known risk factor for SH as a result of a pulmonary and systemic inflammatory response, with its prevalence ranging between 60 and 89% in different studies (15, 18). Vasoconstrictors and inotropic agents such as epinephrine, norepinephrine, and dopamine, along with glucocorticoids, are among the most common medications which are associated with SH, enhancing glycogenolysis, gluconeogenesis, and insulin resistance (19, 20). The excess of glucose administration, either *via* dialysate solutions used in peritoneal dialysis or in the form of parenteral nutrition and intravenous glucose solutions often results in SH, and recent policies suggest the administration of low-calorie parenteral nutrition as a preventive measure (21). Lastly, cardiac surgery has been associated with SH to an incidence that may even exceed 50% in different reports (22, 23).

## Type 1 Diabetes Mellitus: Prediction Models and the Association With Stress Hyperglycemia

### Prediction of T1DM: Current Notions and Prospects

Type 1 diabetes mellitus is a chronic disease where the combination of genetic predisposition and environmental factors induce β-cell autoimmunity, which leads to insulin depletion. During the recent decades, a primary research target has been the creation of predictive models which would incorporate genetic and immune markers, environmental factors, and other variables to accurately identify children at the greatest risk of the disease (6). Below, the main variables included in such models will be briefly described.

### Genetics

T1DM does not have a steady and clear inheritance pattern. Among the patients with T1DM, only 13% have a first-degree relative with the disease (24); similarly, in children without a family history of T1DM, the risk ranges from 0.01 to over 5% (25). Susceptibility to the disease has been associated with over 60 genetic loci, yet alleles at the HLA DR and HLA DQ class II loci present the strongest association (26). The HLA DRB1^*^03,^*^04; DQB1^*^0302 genotype is present in 39% of patients who develop T1DM before age 20, providing a risk of 6.8%, the highest among all genotypes (27). However, such risk ratios are also affected by other factors such as family history, while it should be noted that there are also protective combinations, such as HLA DQB1^*^0602 (28).

### Autoantibodies and Novel Biomarkers

The emergence of autoantibodies signals the initiation of the autoimmune destruction of beta cells. Four primary types of islet autoantibodies are detected: against GAD 65, insulin (IAA), insulinoma antigen-2 (IA-2), and zinc transporter 8 (ZnT8) (29). The presence of only one autoantibody is associated with a minimal increase in the risk of overt disease, referring only to the 10% of total patients. On the contrary, the risk of type 1 diabetes increases as the number of relevant autoantibodies detected increases, with 70% of diabetic children having at least three autoantibodies developing overt T1DM within 10 years (30). Apart from their number, other qualitative factors such as titer levels and affinity affect the risk for T1DM, and IA-2 autoantibodies are associated with the highest risk (6). Currently, the measurement of autoantibodies is the most feasible approach in those individuals who are considered genetically prone to the disease. Novel approaches include transcriptomics (study of patterns of gene expression) in autoantibody-positive children and metabolomics, where levels of certain lipids such as phosphatidylcholines act as prediction markers before seroconversion; however, such procedures cannot be used in everyday clinical practice (26, 31).

### Environmental and Other Factors

The most firmly established factor is the presence of family history (32, 33), with a recent study by Awadalla et al. (34) demonstrating an odds ratio (OR) of 9.03 in children with fathers with T1DM. However, external factors that contribute to the development of T1DM also include nutrition, viral infections, birth weight, maternal age, increased childhood growth, perinatal conditions, and geographic diversity (34).

Breastfeeding and the introduction to cow's milk have long been a matter of debate. On the one hand, Mayer et al. (35) reported that breastfeeding for more than 12 months reduced the risk of T1DM (OR:0.54), while other studies showed that breastfeeding for <6 months or not at all importantly increased the risk for T1DM by at least 30% (36–39). On the other hand, several reports showed only a very weak inverse association between breastfeeding and T1DM and only in mothers aged over 35 years old, or even no association at all (40, 41). Even more interestingly, a recent meta-analysis of 43 studies including 9.874 patients with T1DM showed only a weak protective effect of exclusive breastfeeding for >2 weeks, however, results were subject to marked heterogeneity (42). Overall, most research data seem to favor exclusive and prolonged breastfeeding.

Perinatal conditions are important determinants of future T1DM development. In a meta-analysis of 20 observational studies, the risk of T1DM was higher in children born by cesarean section (OR: 1.23); the mechanisms are yet to be elucidated (43). A positive association of maternal age and T1DM risk was also identified; this finding has been confirmed in a meta-analysis by Cardwell et al., where there was a 5% increase in T1DM odds per 5-year increase in maternal age (44). Birth weight is another important variable. In a population-based cohort study on 1,824 children with T1DM (45), the rate ratio for children with birth weights ≥ 4,500 g compared with those with birth weights ≤ 2,000 g was 2.21, similarly to previous works by Dahlquist et al. (46), where children who were large for gestational age (LGA) were at increased risk for type 1 diabetes (47). On the other hand, in a twin-control study by Kyvik et al. (48), no relationship between T1DM and birth weight was established, and this was the case also in the study by Bock et al. (49) and other previous reports (47). Despite the inconsistency of the results, the association between birth weight and T1DM cannot be ignored; other factors, such as pre-eclampsia, maternal obesity, and stress have also been associated with the development of diabetes (50).

Cytomegalovirus, rubella, and enteroviruses have all been associated with T1DM (51, 52). Low levels of serum 25-OH vitamin D among patients with T1DM compared to healthy controls have been shown (53, 54); however, in a study by Bierschenk et al., no such association was found (55). It is noteworthy that in the former studies, the setting was largely northern Europe, while the latter was performed in a solar-rich environment, implying a geographic component in the course of T1DM (56). The data about the association between rural residence and T1DM, however, has been contradictory (57, 58).

## Stress Hyperglycemia as a Predictive Factor of T1DM

Whether stress hyperglycemia could be a trigger factor for overt T1DM is not clear. As non-diabetic patients are able to compensate insulin resistance by increasing insulin secretion, it seems logical to assume that the presence of stress hyperglycemia could imply some degree of pre-existing β-cell dysfunction. In addition, hyperglycemia in the context of acute illness provokes the overexpression of the insulin-independent glucose transporters GLUT-1, GLUT-2, and GLUT-3, which leads to excessive glucose uptake and, thus, to increased glycolysis and oxidative phosphorylation in peripheral tissues (1, 3). These actions lead, in turn, to enhanced production of reactive oxygen species (ROS) and, therefore, increased oxidative stress, which stimulates the classic intracellular pathways that are involved in T1DM pathogenesis: the polyol pathway, the activation of protein kinase C (PKC), the increased intracellular production of advanced glycation end products (AGEs), and the hexosamine pathway (1, 59). The consequent lipotoxicity, immune dysregulation, endothelial dysfunction, and hyperinflammatory response with increased cytokine production could theoretically comprise a causative inflammatory pathway that may lead to type 1 diabetes (60–62).

Stress hyperglycemia is a well-established risk factor for type 2 diabetes in adults. In the study by Plummer et al. (63), among 2,883 patients with stress hyperglycemia, the incidence of type 2 diabetes following critical illness was 4.8%, and the risk of diabetes in these patients compared to controls was almost two-fold (HR: 1.91); similarly, McAllister et al. (64) showed 3-year risk of developing type 2 diabetes of 2.3% in patients with SH, and a positive association of this risk and degree of SH was noted. However, research data in children and their risk of incident T1DM has been inconsistent (Table 1). Early reports have been in favor of this notion, at least in part. Vardi et al. (65) studied 12 children with stress hyperglycemia and a mean age of 7.2 ± 4.5 years and found that 58% of the children had a positive autoimmune assay, while 33% of the children progressed to overt T1DM within a year of follow-up, established by a low first-phase insulin release during an intravenous glucose tolerance test (IVGTT). Herskowitz et al. found that 7/63 (11%) of patients with transient hyperglycemia developed T1DM within one year, although it was noted that the risk was associated with disease severity (66). More specifically, T1DM was identified in 32% of children in whom transient hyperglycemia was discovered in the absence of a serious illness, compared with 2.3% of children identified during a serious illness. However, it is acknowledged that the short duration of the follow-up period in these studies may have underestimated the risk for overt T1DM in the future. In a prospective study by Schatz et al. (67), where 29 children with stress hyperglycemia or glycosuria were examined, all five subjects with islet cell antibodies or human leukocyte antigen DR3/DR4 eventually developed overt diabetes or had abnormal glucose tolerance within 8 years of follow-up. This was demonstrated, however, only in one out of the five children who lacked these biomarkers, suggesting that in the absence of ICA and HLA-DR3/DR4 heterozygosity, transient hyperglycemia is unlikely to develop to overt diabetes. Similarly, in a study in Iran, 50 children with a mean age of 9.81 ± 1.45 years and a body mass index (BMI) of 21.7 ± 3.7 kilograms/m^2^, who were hospitalized due to stress hyperglycemia, were studied for the presence of metabolic syndrome or GAD65 autoantibodies. Although no cases of overt T1DM were found, the prevalence of insulin resistance, measured by the Homeostatic Model Assessment for Insulin Resistance (HOMA-IR), was 16% during follow-up (68). However, the most robust association between SH and T1DM was established in a multicenter Italian study on 748 subjects with a mean age of 9.04 ± 3.62 years and without a family history of diabetes, where immunological (ICAs, IAAs, and GADAs), metabolic (first-phase insulin response-FPIR), and immunogenetic (serological HLA typing for class I and class II and molecular analysis of HLA-DQA1 and -DQB1 genes) markers were examined (69). It was shown that islet cell autoantibodies were present in 10% of the children with SH, elevated insulin autoantibody levels in 4.6%, GAD antibody in 4.9%, and anti-tyrosine phosphatase-like protein autoantibodies in 3.9%, while also the HLA-DR3/DR3 and HLA-DR4/other alleles were more frequent in these patients. First-phase insulin response (FPIR) was diminished in 25.6% of subjects during an oral glucose tolerance test (OGTT) and, eventually, after a median follow-up of 42 months, 2.1% of subjects developed overt disease. It must be noted, however, that the transient hyperglycemia of the children enrolled was found without serious intercurrent illness once again.

**Table 1:** Major studies on the association between stress hyperglycemia and incidence of type 1 diabetes mellitus.

**Authors**	**Patients (n)**	**Mean age (y)**	**Follow-up duration**	**Primary acute condition**	**Association with T1DM**
Vardi et al. (65)	12	7.2 ± 4.5	4.75 + 4.3 months	RTI, fever, abdominal pain, meningitis, and cellulitis	33% progressed to overt diabetes
Herskowitz et al. (66)	63	7.4 ± 4.8	2.9 ± 1.9 years	two groups: without acute illness vs. RTI, UTI, meningitis, gastroenteritis, seizures, surgery	32% without acute condition vs. 2.3% with acute illness
Schatz et al. (67)	29	9.2 ± 5.8	2 years ± 9 months	N/A—Referral for hyperglycemia or glycosuria	5/5 patients with ICA (+) or HLA DR3/DR4 (+)−1/5 patients with (-) markers
Eshraghi et al. (68)	50	9.8 ± 1.4	N/A	Not mentioned	No cases of T1DM−16% IR
Lorini et al. (69)	748	9.04 ± 3.62	42 months	N/A—incidental hyperglycemia	10% ICA (+), 4,6% IAA (+), 4,9% GADA (+), 3,9% IA-2A (+), 2,1% overt disease during follow-up
Bhisitkul et al. (70)	90	2	30-36 months	Sickle cell disease, gastroenteritis, RTI, seizures, injury	No association
Shehadeh et al. (71)	36	6.2	3.2 years	Gastroenteritis, febrile seizures, pneumonia	No association
Valerio et al. (12)	1,199	5.2 ± 4.5	3.5 ± 0.6 years	Febrile seizures, traumatic injuries, appendicitis, abdominal pain, gastroenteritis, febrile infections	No association
Bordbar et al. (72)	1,054	1.5	24 months	Gastroenteritis, seizures, pneumonia, sepsis, poisoning, UTI	No association
Weiss et al. (73)	55,120 patient visits	5.7	4-10 years	RTI, trauma, seizures	No association

*RTI, respiratory tract infection; UTI, urinary tract infection; N/A, not applicable; ICA, islet cell antibodies; HLA, human leukocyte antigen; IAA, insulin autoantibodies; IA-2A, protein tyrosine phosphatase antibodies; GADA, glutamic acid decarboxylase antibodies*.

On the other hand, several reports have risen serious doubts as to whether SH can predispose to T1DM either by itself or in combination with other factors. In a study by Bhisitkul et al. (70), all children who were evaluated for an acute illness or injury were prospectively screened for hyperglycemia. Blood samples were obtained from 30 hyperglycemic children, 30 stress control subjects, and 30 healthy control subjects. It was shown that, in the absence of autoantibodies or genotypes at the DQB1 gene, none of the hyperglycemic subjects progressed to diabetes within 36 months. Similar were the results in a study by Shehadeh et al. (71) on 36 children with SH during severe intercurrent illness. Insulin autoantibodies were present only in three subjects and, despite the low first-phase insulin response in eight patients during the initial evaluation, results were normal after 12-16 months. More importantly, no patient developed T1DM after 3.2 years of follow-up. In more recent reports, Valerio et al. (12) showed that, in 41 children with SH and no family history of diabetes, none developed diabetes after a 3.5 ± 0.6-year period of follow-up. In a prospective cross-sectional study by Bordbar et al. (72), none of the 39 children with SH, evaluated with fasting blood glucose measurements with 6-month intervals, were diagnosed with T1DM during the 2-year follow-up. As it was noted by the researchers, however, a greater follow-up period might be necessary to confirm whether SH is a first sign of T1DM. This need was addressed in a retrospective cohort of 55,120 consecutive visits over 6 years to a pediatric emergency department where 72 cases of extreme SH (>300 mg/dl) were identified; no cases of T1DM were found among the patients who were followed-up for 4-10 years (84%) (73).

## Conclusion

Stress hyperglycemia is a normal homeostatic response to acute stress, which is characterized by increased glycogenolysis and gluconeogenesis along with insulin resistance. It has a high prevalence in the pediatric population, with febrile conditions, respiratory infections and medications being among the most common causes. However, apart from its protective effects, its significance as a warning sign for future progression to T1DM has been a matter of research and controversy. T1DM is a disease with a clear genetic origin, but genetic screening cannot easily be applied to the general population and, even more importantly, genetic susceptibility alone is not sufficient to predict whether a child will ever develop the overt disease. Therefore, antibody testing and environmental factors such as breastfeeding, maternal age, delivery by cesarean section, birth weight, and viral infections have been under the spotlight as possible components of diagnostic algorithms and predictive models which can readily be available on a broad scale. Research data, albeit relatively scarce, have shown that stress hyperglycemia *per se* cannot be considered an accurate risk factor for future T1DM and, therefore, screening methods including antibody testing or oral glucose tolerance tests are not indicated in children and adolescents presenting only with this clinical manifestation and without other risk factors. However, it seems that, when combined with other risk factors such as a positive family history of T1DM and the presence of autoimmunity, stress hyperglycemia can indeed be the very first sign of disrupted β-cell function and, thus, a part of a broader prediction model which can be applied to genetically prone children and distinguish those individuals who are at greatest risk and, thus, in need of a tighter and more prolonged follow-up.

## Author Contributions

TA, EK, and VL performed the literature review and drafted the manuscript. AG, AK, SK-N, and AR provided guidance and edits. All authors read and approved the final manuscript.

## Conflict of Interest

The authors declare that the research was conducted in the absence of any commercial or financial relationships that could be construed as a potential conflict of interest.

## Abbreviations

ADA: American Diabetes Association
AGE: advanced glycation end product
BMI: body mass index
FPIR: first-phase insulin response
GAD-65: glutamic acid decarboxylase
GH: growth hormone
GLUT: glucose transporter protein
HLA: human leukocyte antigen
HOMA-IR: Homeostatic Model Assessment for Insulin Resistance
HPA: hypothalamic-pituitary-adrenal
IA-2A: protein tyrosine phosphatase antibodies
IAA: insulin autoantibodies
ICU: intensive care unit
IGF-1: insulin-like growth factor-1
IL-1: interleukin 1
IL-6: interleukin 6
IVGTT: intravenous glucose tolerance test
MODY: maturity-onset diabetes of the young
OGTT: oral glucose tolerance test
PKC: protein kinase C
ROS: reactive oxygen species
SH: stress hyperglycemia
T1DM: type 1 diabetes mellitus
T2DM: type 2 diabetes mellitus
TNF-α: tumor necrosis factor α
ZnT8: zinc transporter 8.
